# Super-resolution reconstruction based on Gaussian transform and attention mechanism

**DOI:** 10.7717/peerj-cs.1182

**Published:** 2023-01-12

**Authors:** Shuilong Zou, Mengmu Ruan, Xishun Zhu, Wenfang Nie

**Affiliations:** 1 Nanchang Normal College of Applied Technology, School of Electronic and Information Engineering, Nanchang, Jiangxi, China; 2 Nanchang Institute of Science & Technology, School of Wealth Management, Nanchang, Jiangxi, China; 3 Current Affiliation: School of Economics and Management, Jiangxi Manufacturing Polytechnic College, Nanchang, China

**Keywords:** Super-resolution reconstruction, Multi-scale, Gaussian difference transform, Attention mechanism

## Abstract

Image super-resolution reconstruction can reconstruct low resolution blurred images in the same scene into high-resolution images. Combined with multi-scale Gaussian difference transform, attention mechanism and feedback mechanism are introduced to construct a new super-resolution reconstruction network. Three improvements are made. Firstly, its multi-scale Gaussian difference transform can strengthen the details of low resolution blurred images. Secondly, it introduces the attention mechanism and increases the network depth to better express the high-frequency features. Finally, pixel loss function and texture loss function are used together, focusing on the learning of structure and texture respectively. The experimental results show that this method is superior to the existing methods in quantitative and qualitative indexes, and promotes the recovery of high-frequency detail information.

## Introduction

Image super-resolution (SR) reconstruction is a basic task of image processing and is widely used in image compression (Chen et al., 2021a; Shi, Li & Jiahuan, 2022; Chen et al., 2021b), medical imaging (Jiao et al., 2020), and other fields. It is a research hotspot in the field of image processing. Current SR methods can be divided into two categories: reconstruction-based and learning-based methods.

Reconstruction-based methods can be divided into two categories: frequency domain methods and spatial domain methods. Image reconstruction based on the frequency domain (transform domain) is the indirect processing of images directly in the transform domain. The transformations used include Wavelet transform (Jing & Wu, 2014), Fourier transform (Liu, 2018), etc. (Xiong et al., 2015). These algorithms are simple in principle, have low computational complexity, and are easy to implement in hardware. Therefore, the frequency methods can only deal with global translational motion and cannot utilize the image a priori information.

The spatial domain methods mainly include iterative back-projection (IBP) (Irani & Peleg, 1991; Song et al., 2010; Seema & Bailey, 2019), projections onto convex sets (POCS) (Patti & Altunbasak, 2001; Fan et al., 2017; Ma & Ren, 2020), maximum a posteriori (MAP) probability estimation method (Shen et al., 2007; Belekos, Galatsanos & Katsaggelos, 2010; Nascimento & Salles, 2020) and other methods. These methods use the sub-pixel information existing between low-resolution (LR) images to provide additional information for reconstructing the images. The algorithms reconstruct well and are mainly used in scientific research, satellite remote sensing and other fields. However, these classical reconstruction methods require researchers to have a large amount of a priori knowledge and deep professional reserves. With the rise of deep learning, learning-based reconstruction methods have received wide attention because the technology does not require much a prior knowledge and the quality of reconstructed images is better than that of traditional algorithms.

Convolutional neural network (CNN) is one of the main algorithms for deep learning, and has excellent performance in areas such as image classification (Wang, Wang & Wang, 2015) and computer vision (Gatys, Ecker & Bethge, 2015a). Super-resolution convolutional neural network (SRCNN) was proposed by Dong et al. (2014) to apply CNN to super-resolution reconstruction. The proposed SRCNN is of milestone significance, but there are some shortcomings, such as over-reliance on contextual information of small image areas and slow convergence speed during training. In view of these shortcomings, Dong, Loy & Tang (2016) proposed the fast super-resolution convolutional neural network (FSRCNN). Increasing the number of network layers and using a smaller convolution kernel make the network deeper and learn more features. Kim, Lee & Lee (2016) proposed very deep convolutional networks for super-resolution (VDSR) models with increased number of network layers.

Deepening the network structure can bring more features, but it also tends to cause gradient disappearance or gradient explosion. ResNet (He et al., 2016) can effectively solve these problems and improve the expressiveness of the network. Subsequently, Mao, Shen & Yang (2016) applied it to the very deep residual encoder–decoder network (RED-Net). Unlike ResNet, DenseNet (Huang et al., 2017) connects each layer in series with other layers, which can better preserve the characteristic information of the original image. Even if it is transferred to the later layers, the image information is not easily lost, and the problem of gradient disappearance is well solved. Considering the advantages of DenseNet, Tong et al. (2017) proposed SRDenseNet. Zhang et al. (2018b) combined the residual block with the dense module to form the residual dense block (RDB). Yu et al. (2018) proposed the Wide Activation Image Super-Resolution (WASR) model. WASR consists of a convolution module, a residual module, and a pixel reorganization module. The pixel shuffle is realized by using the sub-pixel layer proposed by the efficient sub-pixel convolutional neural network (ESPCN) (Shi et al., 2016) for up sampling.

The images generated by these algorithms have a high peak signal-to-noise ratio (PSNR), but the perceptual quality is poor. To generate images for human eye perception, Ledig et al. (2016) proposed to realize image super-resolution by means of a Generative Adversarial Network (SRGAN). The main body of the network adopts a Generative Adversarial Network (GAN) (Goodfellow et al., 2014). Although the PSNR is not the highest, the reconstructed image is more natural and clear, which is more in line with the visual effect of human eyes. Wang et al. (2018b) improved SRGAN and proposed Enhanced super-resolution Generative Adversarial Networks (ESRGAN). SRGAN uses activated features to calculate perceptual loss, while ESRGAN uses pre-activated features to calculate perceptual loss. Wang, Yu & Dong (2018) proposed SFTGAN and added a new spatial feature transform (SFT) to the model that combines effective a priori information with the neural network for end-to-end training. The reconstructed images appear visually more natural.

Most of the neural network-based SR methods do not fully utilize the information of the original LR image, resulting in unsatisfactory results. Zhang et al. (2018a) proposed the residual channel attention network (RCAN) by combining the channel attention mechanism with the residual block. Zhang et al. (2018a) also pointed out that there is a large amount of low-frequency information in low-resolution images, which can be transmitted directly to the last layer of the network through long-hop connections, allowing the network to focus on learning high-frequency information and reducing the learning burden of the network.

In addition to improving the network structure, different loss functions are used to generate different image qualities. Commonly used loss functions include pixel loss, content loss, confrontation loss (Mao et al., 2017), texture loss (Gatys, Ecker & Bethge, 2015b; Gatys, Ecker & Bethge, 2016), total variation loss (Rudin, Osher & Fatemi, 1992), and context loss (Mechrez et al., 2018). Since each loss function has its own emphasis, combining multiple loss functions to train the network, the image generated by the model will achieve good results in both objective evaluation and subjective visual effects. At present, most models adopt a joint training method with multiple loss functions, such as EnhanceNet proposed by Sajjadi, Scholkopf & Hirsch (2017). The loss function consists of perceptual loss, confrontation loss, and texture loss, which can produce a realistic texture.

In order to generate realistic textures and natural details with high visual quality, this paper combines reconstruction methods and learning methods to build a deep learning network Garesat-Net based on Gaussian transform, residuals and channel attention. In the network, a new detail enhancement module based on Gaussian transform is constructed. The new module and the channel attention mechanism module are embedded into the residual structure to form a new residual block. In the network, the residual block is used to enhance the details of the image and send it to the subsequent layers for training. During the training process, the loss function comprises pixel loss and texture loss to limit the generation of super-resolution images. Through simulation experiments and real data testing, our algorithm can effectively utilize color image channel correlation and outperform existing algorithms in reconstruction.

The rest of the paper is organized as follows. The related work and evaluation metrics are given in the next section. Section 3 describes the workflow of the proposed model in detail. To evaluate the proposed algorithm, simulation and experimental results are given in Section 4. Finally, a brief conclusion is drawn in Section 5.

## Related Work and Evaluation Indexes

### Multi-scale Gaussian difference transform

Gaussian smoothing is a kind of digital image processing method, calculated from a two-dimensional normal distribution (Gaussian distribution) function. The Gaussian kernel used in the two-dimensional Gaussian distribution function is the product of two one-dimensional Gaussians x and y, and the standard deviation *σ* isusually the same in both dimensions:

(1) \begin{eqnarray*}G(x,y)= \frac{1}{2\pi {\sigma }^{2}} {e}^{ \left( - \frac{{x}^{2}+{y}^{2}}{2{\sigma }^{2}} \right) }.\end{eqnarray*}

Gaussian smoothing is the convolution of an image with a two-dimensional Gaussian kernel of a certain size. Gaussian kernel is a discrete approximation of a continuous Gaussian function, which is usually obtained by discrete sampling and normalization of Gaussian surfaces. Here, normalization means that the sum of all elements of the convolution kernel is 1. The standard deviation *σ*determines the influence of the surrounding pixels on the current pixel. When *σ* increases, the influence of distant pixels on the central pixel is improved, with smoother filtering results. The Gaussian kernel is a discrete approximation of the continuous Gaussian. The more natural the window is, the better the approximation is. However, the Gaussian function is a bell-shaped curve. The farther away from the center, the smaller the value, which can be ignored if the distance is far enough. After the standard deviation is determined, the radius is 3*σ*, that is, the window size is 6*σ* × 6*σ*. Usually, the nearest odd number is taken.

The Gaussian multi-scale difference method (Kim et al., 2015) uses different Gaussian kernel functions to obtain different Gaussian blurred images, so as to extract different detailed images and fuse different detailed images into a whole. First, different Gaussian blurred images are obtained using different Gaussian kernel functions, and the image to be enhanced is I:

(2) \begin{eqnarray*}{B}_{1}(I)={G}_{1}\ast I,{B}_{2}(I)={G}_{2}\ast I,{B}_{3}(I)={G}_{3}\ast I\end{eqnarray*}

where *G*_*k*_k =1 (, 2, 3) is the Gaussian kernel function. We extract fine detail *D*_1_, intermediate detail *D*_2_ and rough detail *D*_3_, which are given by the following formula:

(3) \begin{eqnarray*}{D}_{1}(I)=I-{B}_{1}(I),{D}_{2}(I)=I-{B}_{2}(I),{D}_{3}(I)=I-{B}_{3}(I).\end{eqnarray*}

Merging the three-layer detail map to generate the overall detail and adding it to the original image:

(4) \begin{eqnarray*}{I}^{\ast }=I+(1-{\omega }_{1}\times \mathrm{sgn}({D}_{1}(I)))\times {D}_{1}(I)+{\omega }_{2}\times {D}_{2}(I)+{\omega }_{3}\times {D}_{3}(I).\end{eqnarray*}

where *ω*_1_, *ω*_2_, *ω*_3_are the weight coefficients and sgn is a step function.

**Figure 1:**
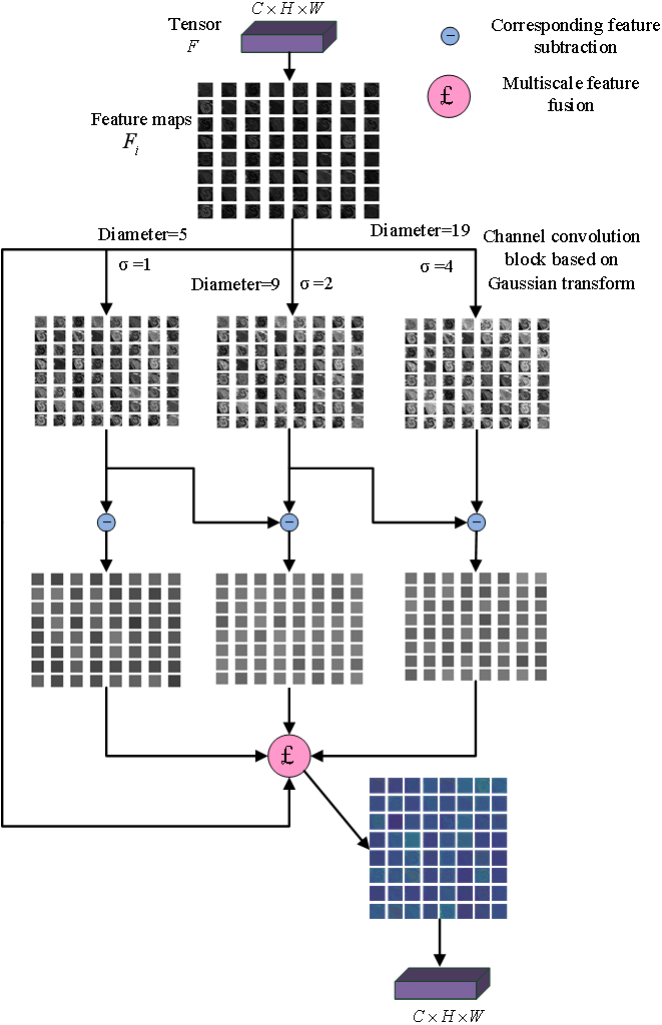
Gaussian enhancement block.

Gaussian multi-scale difference method is introduced in CNN to construct a Gaussian enhancement block, as shown in Fig. 1. The input multi-channel features are convoluted by a three-channel convolutional layer based on different Gaussian kernels, respectively, and three different Gaussian fuzzy feature maps are obtained. Three detail maps were obtained by making a circular difference with the original feature maps, and then fused with the original feature map to obtain the enhanced feature map. The parameters of fusion *ω*_1_, *ω*_2_, *ω*_3_were fixed at 0.5, 0.5 and 0.25.

### The attention mechanism

Humans can naturally and effectively find significant regions in complex scenes. Inspired by this observation, an attention mechanism was introduced into computer vision to imitate this aspect of the human visual system. This attention mechanism can be viewed as a dynamic weight adjustment process based on the input image features, which is widely used in various types of deep learning tasks such as natural language processing, image recognition and speech recognition. It is one of the core technologies that deserve attention and deeper understanding in deep learning technology. The core goal of deep learning attention mechanism is to select more critical information for the current task from a large amount of information. Its representatives are channel attention and spatial attention.

#### Channel attention block

**Figure 2:**
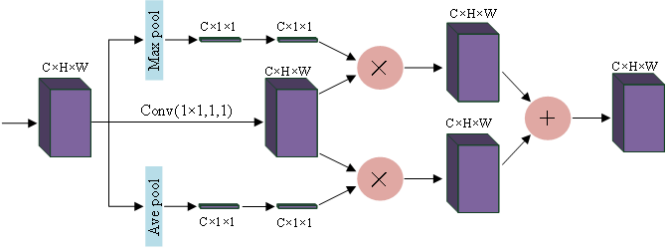
Channel attention block.

Squeeze-and-Excitation Networks (SENet) (Jie, Li & Gang, 2018) considered the relationship between feature channels and an added attention mechanism to the feature channels. SENet automatically obtains the importance of each feature channel through learning and uses the obtained importance to improve features and suppress features that are not important for the current task. Using global maximum pooling and average pooling, the spatial dimension is compressed to obtain the channel weights of the different features, and this feature is applied to the original channel, and the features that need to be enhanced are augmented, and the two different enhanced features are fused and output. For more information, please see Fig. 2.

#### Spatial attention block

**Figure 3:**
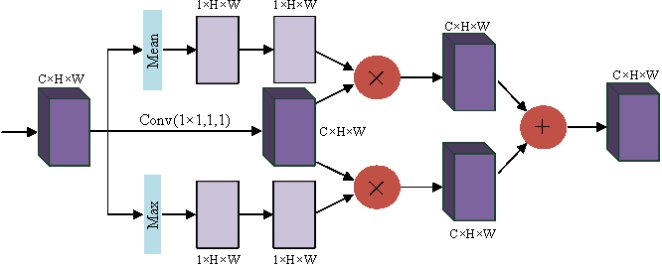
Spatial attention block.

In the convolution layer, the receptive field is limited due to the size limitation of the convolution kernel. Wang et al. pointed out in Wang et al. (2018a) that nonlocal means that the receptive field can be very large, rather than the local field. If global information can be introduced into some layers, the problem of not seeing the global situation for local operations can be solved, bringing richer information to the later layers. A module called spatial transformer is proposed to transform the spatial information in the image so as to extract the key information. The essence of spatial attention is to locate the target and make some changes or obtain weights. As shown in Fig. 3, the target is located by compressing the channels with mean and maximum values to obtain the weights of different parts. Enhanced features are obtained by interacting with the original feature map, and the two enhanced feature maps are combined and convoluted and output.

### Residual convolutional neural network

The residual convolutional neural network adds a jump connection branch (He et al., 2016) where the input feature map is added directly to the output and then activated. The residual network is a good solution to the degradation problem of deep neural networks (Wang et al., 2019). The error signal can be propagated directly to the lower layer without any intermediate weight matrix transformation, thus alleviating the gradient dispersion problem and achieving better reconstruction. On the premise of the same number of layers, the convergence speed of the residual network is faster. In the super-resolution reconstruction proposed in this paper, the Gaussian transform enhancement module and the attention mechanism block are embedded into the residual module to construct a new residual block.

### Evaluation indexes

The objective evaluation indexes of image hiding and recovery networks used in this paper include peak signal-to-noise ratio (PSNR) (Welstead, 1999), and structural similarity index measure (SSIM) (Wang, Bovik & Sheikh, 2004).

## Materials & Methods

### Garesat-Net

**Figure 4:**
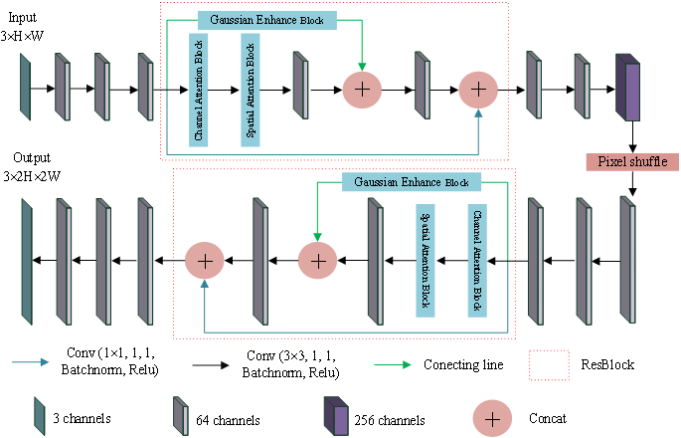
Garesat-Net. The struture of Garesat-Net.

In this section, the super-resolution image reconstruction network, Garesat-Net, was proposed, and the pixel shuffle and the proposed ResBlock were used for this network. The specific structure and parameter settings are shown in Fig. 4. The whole network consists of 11 convolutional layers, two ResBlocks and a pixel shuffle layer. Among them, 10 convolution layers have 64 convolution kernels, and only the sixth convolution layer has 256 convolution kernels, which is to prepare for the next pixel shuffle layer. The ResBlock has three branches. The first branch is the Gaussian enhancement block; the second branch is the channel attention module, as well as the spatial attention module and the convolutional layer, in that order. This output of the second branch is concatenated with the output of the first branch and fed to the next convolutional layer. Finally, the output is concatenated with the output of the third branch. The third branch has only one convolutional layer with a convolution kernel of 1 ×1. The input LR image passes through three convolutional layers, ResBlock, three convolutional layers, pixel shuffle layer, three convolutional layers, ResBlock and three convolutional layers in turn, and the output SR image is twice the length and width of the original image.

### Loss function

During the training process, the loss function combines pixel loss and texture loss to limit the generation of super-resolution images.

#### Pixel loss

Most SR models currently adopt pixel loss. Pixel loss is generally divided into *L*_1_ loss and *L*_2_ loss, and their expressions are as follows:

(5) \begin{eqnarray*}{L}_{1}& = \frac{1}{CHW} \sum _{k=1}^{C}\sum _{i=1}^{H}\sum _{j=1}^{W}{|}{\hat {y}}_{i,j,k}-{y}_{i,j,k}{|}\end{eqnarray*}

(6) \begin{eqnarray*}{L}_{2}& = \frac{1}{CHW} \sum _{k=1}^{C}\sum _{i=1}^{H}\sum _{j=1}^{W}({\hat {y}}_{i,j,k}-{y}_{i,j,k})^{2}\end{eqnarray*}

where C is the number of channels of the image, generally 3; H is the height of the image; W is the width of the image; ${\hat {y}}_{i,j,k}$ is each pixel of the generated high-resolution image; *y*_*i*,*j*,*k*_ is each pixel of the real image.

#### Texture loss

Gatys, Ecker & Bethge (2015b) and Gatys, Ecker & Bethge (2016) introduced texture loss to super-resolution reconstruction. The texture loss uses the Gram matrix. The Gram matrix expression is as follows:

(7) \begin{eqnarray*}{G}_{i,j}^{l}(I)={F}_{i}^{l}(I){F}_{j}^{l}(I)\end{eqnarray*}

where ${F}_{i}^{l}(I)$ is the feature map of the i-th channel on the *l*-th layer of Image *I*; ${F}_{j}^{l}(I)$ is the feature map of the *j* th channel on the *l*-th layer of Image *I*. The expression for texture loss is as follows:

(8) \begin{eqnarray*}{L}_{texture}= \frac{1}{{C}_{l}^{2}} \sqrt{\sum _{i=1}^{{C}_{l}}\sum _{j=1}^{{C}_{l}}({G}_{i,j}^{l}(\hat {y})-{G}_{i,j}^{l}(y))^{2}}.\end{eqnarray*}

The loss function is defined as:

(9) \begin{eqnarray*}\begin{array}{@{}l@{}} \displaystyle L=({L}_{2}+{L}_{texture})/2\\ \displaystyle = \left( \frac{1}{3HW} \sum _{k=1}^{3}\sum _{i=1}^{H}\sum _{j=1}^{W}({\hat {y}}_{i,j,k}-{y}_{i,j,k})^{2}+ \frac{1}{{3}^{2}} \sqrt{\sum _{i=1}^{3}\sum _{j=1}^{3}({G}_{i,j}(\hat {y})-{G}_{i,j}(y))^{2}} \right. \end{array}\end{eqnarray*}

where ${\hat {y}}_{i,j,k},{y}_{i,j,k}$ represent each pixel of SR image and HR image, respectively, $\hat {y},y$ represent SR image and HR image, respectively, and ${G}_{i,j}(\hat {y})-{G}_{i,j}(y)$ represent the Gram matrix of layers i and j of SR image and HR image, respectively. In the training process, pixel loss was used to reconstruct the magnified image that was not fine enough, and texture loss was used to determine the detail part.

### Optimization and parameter initialization settings

The proposed network was optimized using the Adam stochastic optimization method (Kingma & Ba, 2014). The convolution kernel was initialized with Gaussian distribution weights having zero expectation and variance of 0.02. In the experiment, the batch size was 4 and the initial learning rate was set to 0.005. The regularization coefficient in the loss function, the basic learning rate and the weight attenuation were set to 10e−4. After 400 epochs, the loss function became stable without further decline and stopped training.

## Results

In this section, we conducted several experiments to demonstrate the effectiveness of the proposed method.

### Developmental environment and dataset

The proposed Garesat-Net model was trained and tested under a workstation operating system of Windows 10, accelerated with single GeForce RTX 3090Ti graphics adapters. The proposed model is based on Python (3.7) and Pytorch (1.1.0) to build a deep learning model.

As the training datasets, 200 images were used from the Berkeley Segmentation Dataset (BSD)  (Martin et al., 2001). In the testing stage, we employed Set5 (Bevilacqua et al., 2012), Set14 (Zeyde, Elad & Protter, 2010), BSD100 (Martin et al., 2001), and Urban100 (Huang, Singh & Ahuja, 2015) datasets.

### Comparative analysis of related algorithms

This experiment compares the method proposed in this paper with five existing deep learning-based SR algorithms with good performance in this field. The compared methods are RNAN (Zhang, Li & Li, 2019), LatticeNet (Luo, Xie & Zhang, 2020), SCGAN (Liu & Chen, 2021), FPMMLGPR (Lu & Yu, 2022), and AMG (An & Wang, 2022), where the bicubic interpolation method is used as the benchmark algorithm. This comparison verifies the effectiveness and generalization ability of the method proposed in this paper. The trained models published for these methods are used to produce all reconstruction results of the compared methods to ensure the fairness of the experiment. Table 1 shows the PSNR and SSIM indicators obtained for the above five mainstream deep learning-based ISR algorithms on the four test sets.

**Table 1:** Statistical analysis results of the seven methods on the four datasets of Set5, Set14, BSD100 and Urban100.

Methods	Evaluating indicator	Set5		Set14		BSD100		Urban100	
		2	4 ×	2 ×	4 ×	2 ×	4 ×	2 ×	4 ×
Bicubic	PSNR	32.87	28.45	30.25	25.38	29.56	24.80	26.68	22.89
	SSIM	0.9226	0.8777	0.8695	0.8245	0.8441	0.8111	0.8411	0.7905
RNAN	PSNR	36.49	30.89	33.88	28.34	32.29	27.64	32.69	27.66
	SSIM	0.9586	0.8856	0.9213	0.8476	0.9018	0.8398	0.9343	0.8746
LatticeNet	PSNR	36.93	31.11	33.88	28.49	32.30	27.45	32.82	27.94
	SSIM	0.9595	0.8853	0.9213	0.8473	0.9019	0.8367	0.9367	0.8665
SRGAN	PSNR	33.57	30.20	32.89	26.61	32.32	26.61	32.91	27.13
	SSIM	0.9543	0.8726	0.9232	0.7668	0.9021	0.7313	0.9366	0.7742
FPMMLGPR	PSNR	34.83	31.47	33.54	28.59	32.30	26.45	32.54	28.51
	SSIM	0.9527	0.8971	0.9367	0.8464	0.9034	0.8356	0.9367	0.8813
CMG	PSNR	37.14	32.45	33.88	29.69	32.35	**29.88**	32.79	**29.56**
	SSIM	0.9719	**0.9287**	0.9321	0.8888	0.9078	**0.8713**	0.9390	0.8985
Proposed	PSNR	**37.69**	**32.89**	**34.36**	**29.75**	**33.15**	29.54	**33.23**	29.46
	SSIM	**0.9775**	0.9246	**0.9413**	**0.8921**	**0.9162**	0.8691	**0.9411**	**0.9007**

**Notes.**

Bold indicates the best result in this column.

The proposed network reconstructs 2 × super-resolution images and reconstructs 4x and 8x super-resolution images by reuse. The image quality gradually decreases as the number of uses increases. In the following illustration, we show the results of reconstructing 4 × super-resolution images from LR images.

**Figure 5:**
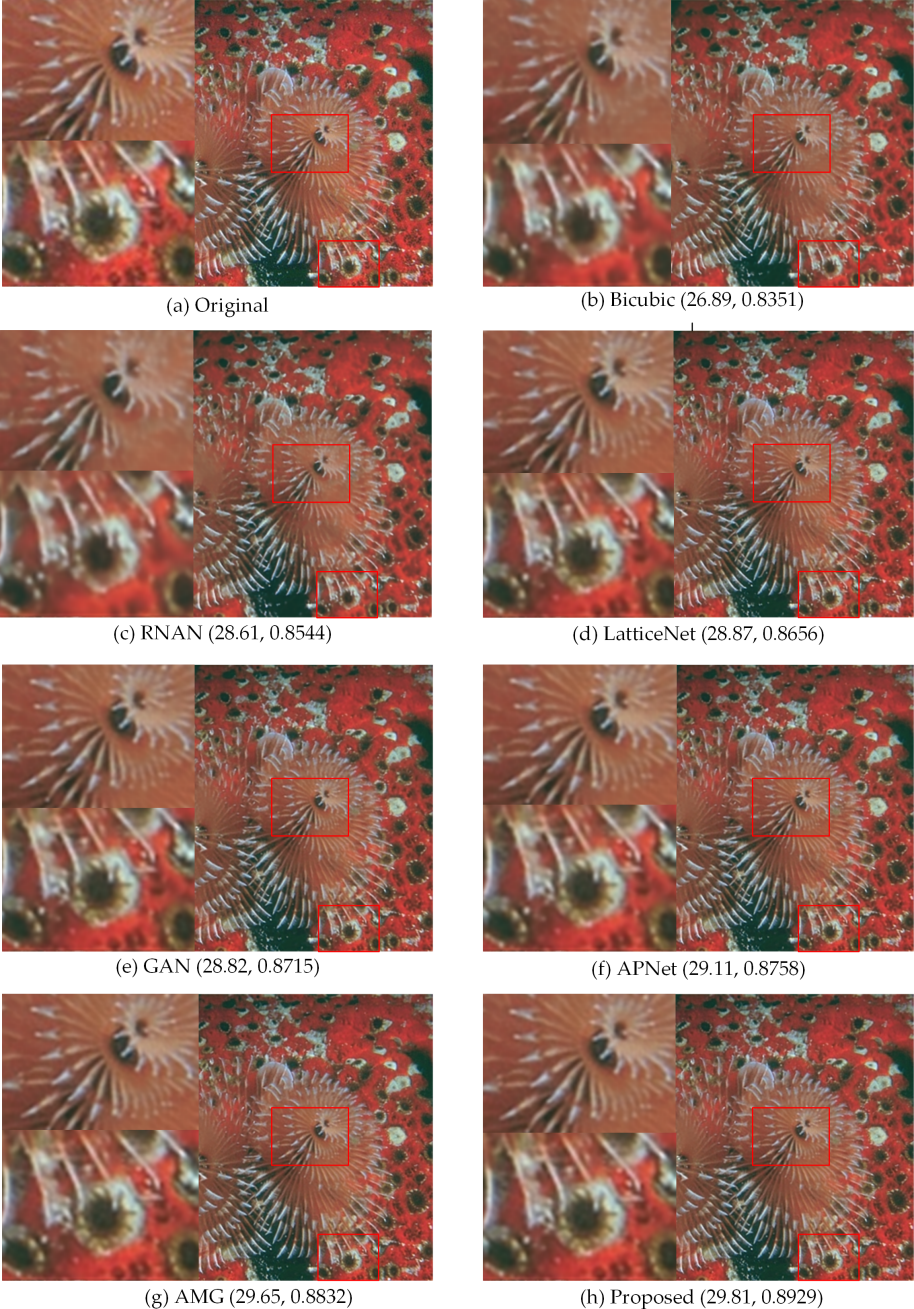
Compared in BSD100. The visual effects produced by different methods of 4 × super-resolution reconstruction are compared in BSD100. Image credit: ©2001 IEEE. Reprinted, with permission, from Martin et al. (2001).

**Figure 6:**
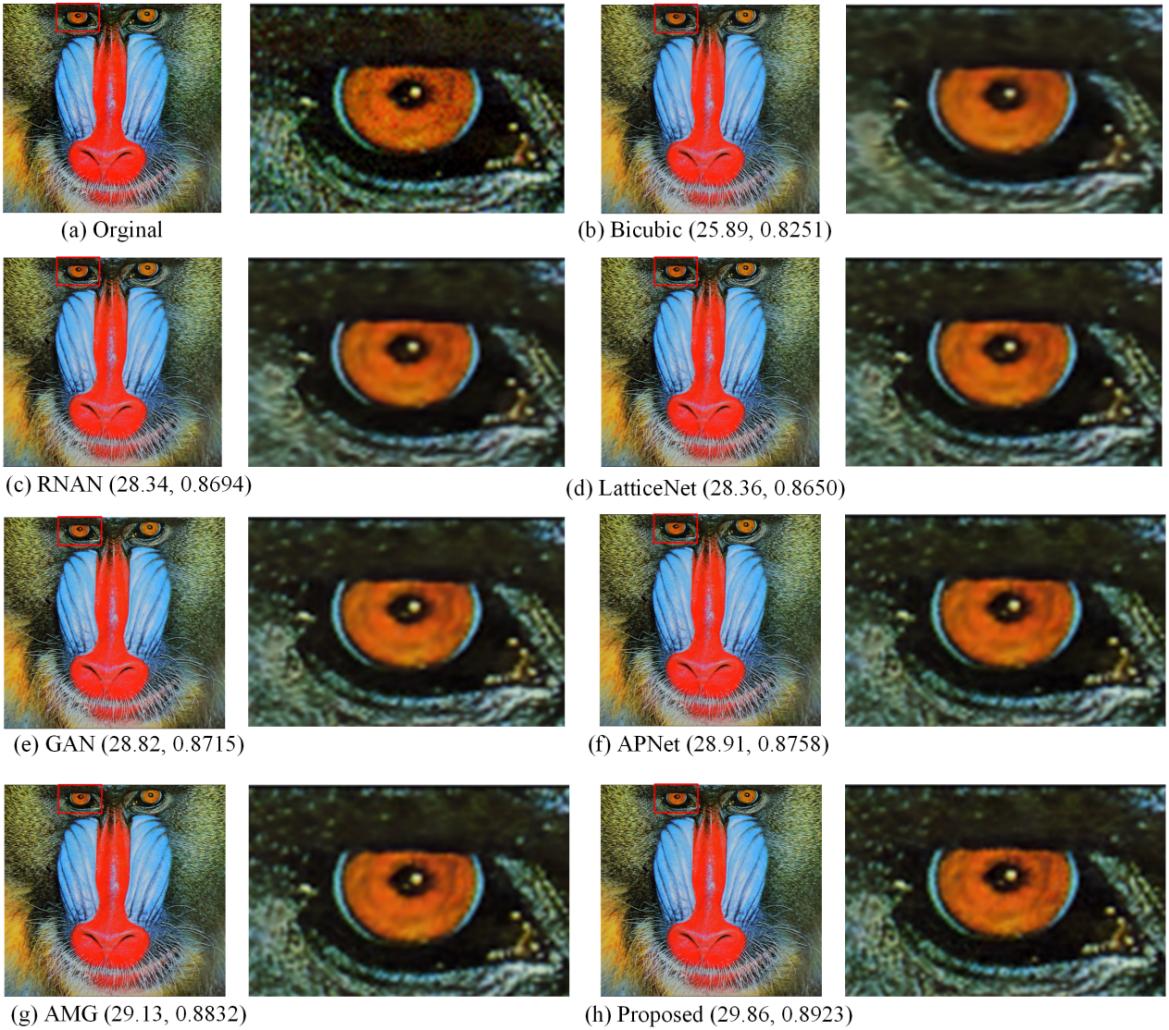
Compared in Set14. The visual effects produced by different methods of 4 × super-resolution reconstruction are compared in Set14. Image source credit: Reprinted by permission from SPRINGER NATURE LICENSE: Springer-Verlag Berlin, Curves and Surfaces (Zeyde, Elad & Protter, 2010). ©2012.

**Figure 7:**
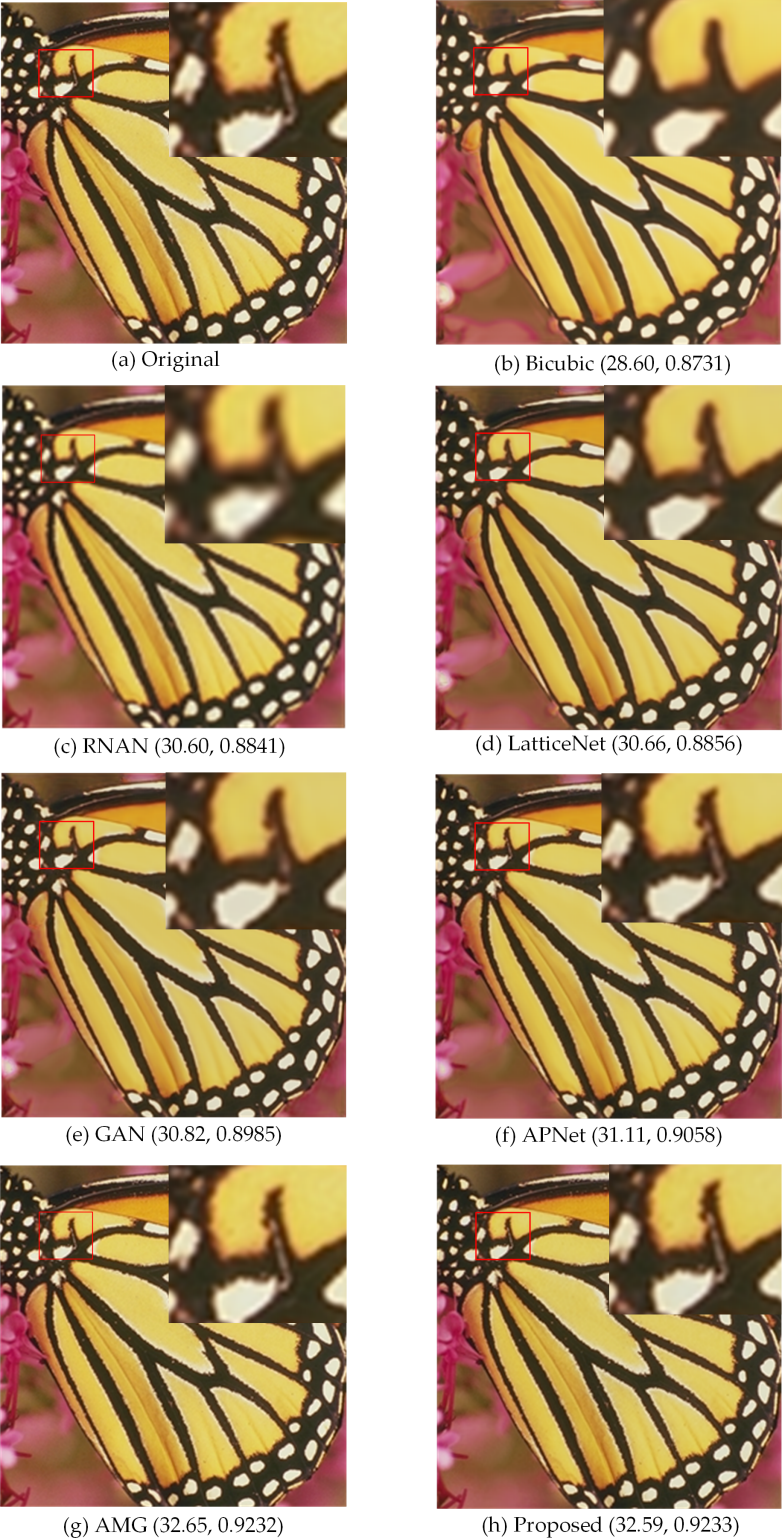
Compared in Set5. The visual effects produced by different methods of 4 × super-resolution reconstruction are compared in Set5. Image source credit: Bevilacqua et al. (2012). Dataset: https://people.rennes.inria.fr/Aline.Roumy//results/SR_BMVC12.html.

**Figure 8:**
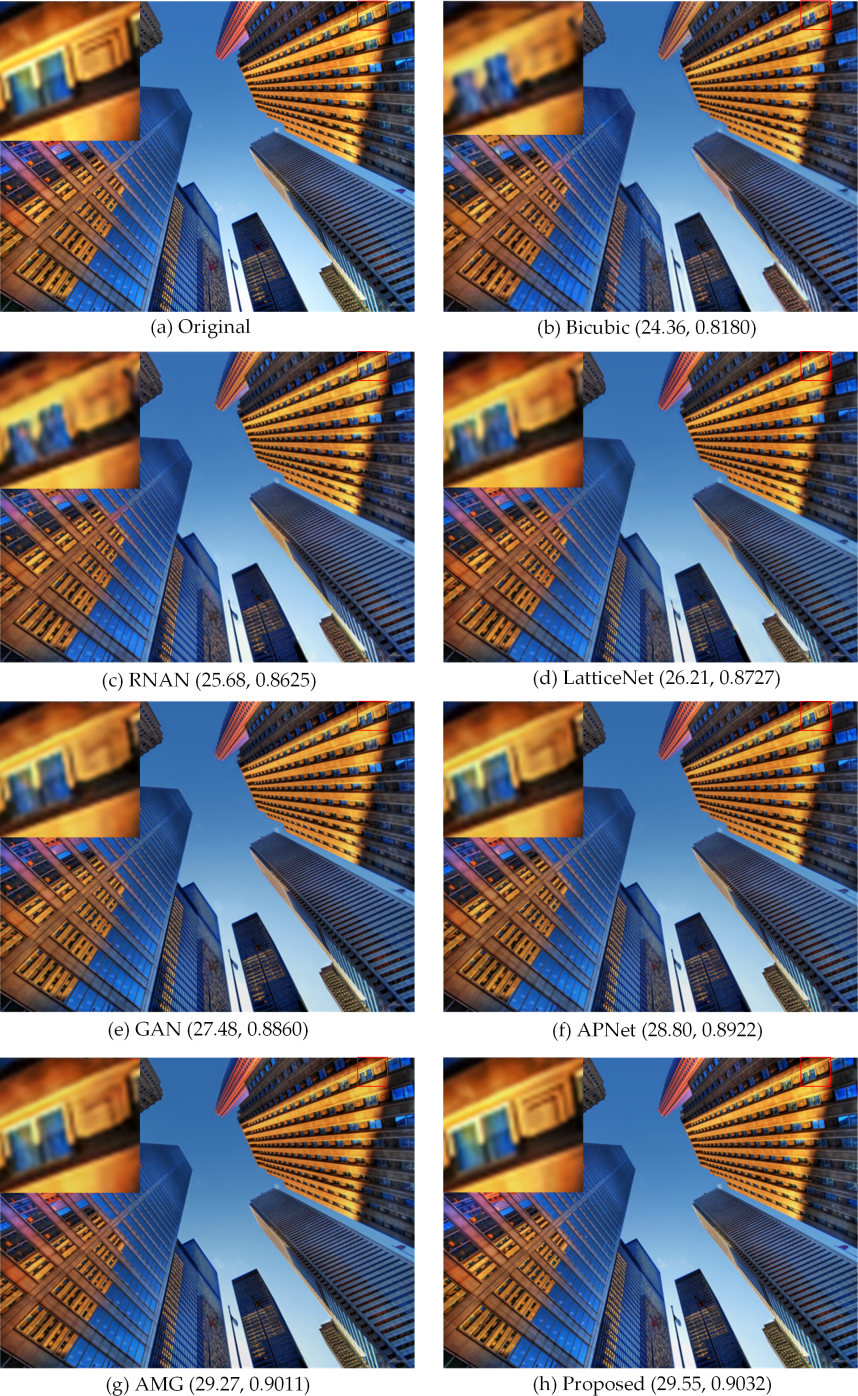
Compared in Urban100. The visual effects produced by different methods of 4 × super-resolution reconstruction are compared in Urban100. Image source credit: ©2015 IEEE. Reprinted, with permission, from Huang, Singh & Ahuja (2015).

Figures 5, 6, 7 and 8 show the visual effects produced by the different 4 × super-resolution reconstruction methods in BSD100, Set5, Set14 and Urban100, respectively. From a simple visual point of view, we cannot tell the difference. When magnifying a part of it, it can be seen that our proposed method is better in terms of texture features and closer to HR images. As can be seen from the spots on the anemone in Fig. 5, upon amplification, the first four and Bicubic are blurred at the edges and have differences in color. Only the AMG method and the proposed method have high quality in terms of reconstructed edges and colors. In Fig. 6, the blood filaments in the baboon’s eyes have been blurred in the reconstruction of the previous methods, and our method only yields similar smoothed results.

**Figure 9:**
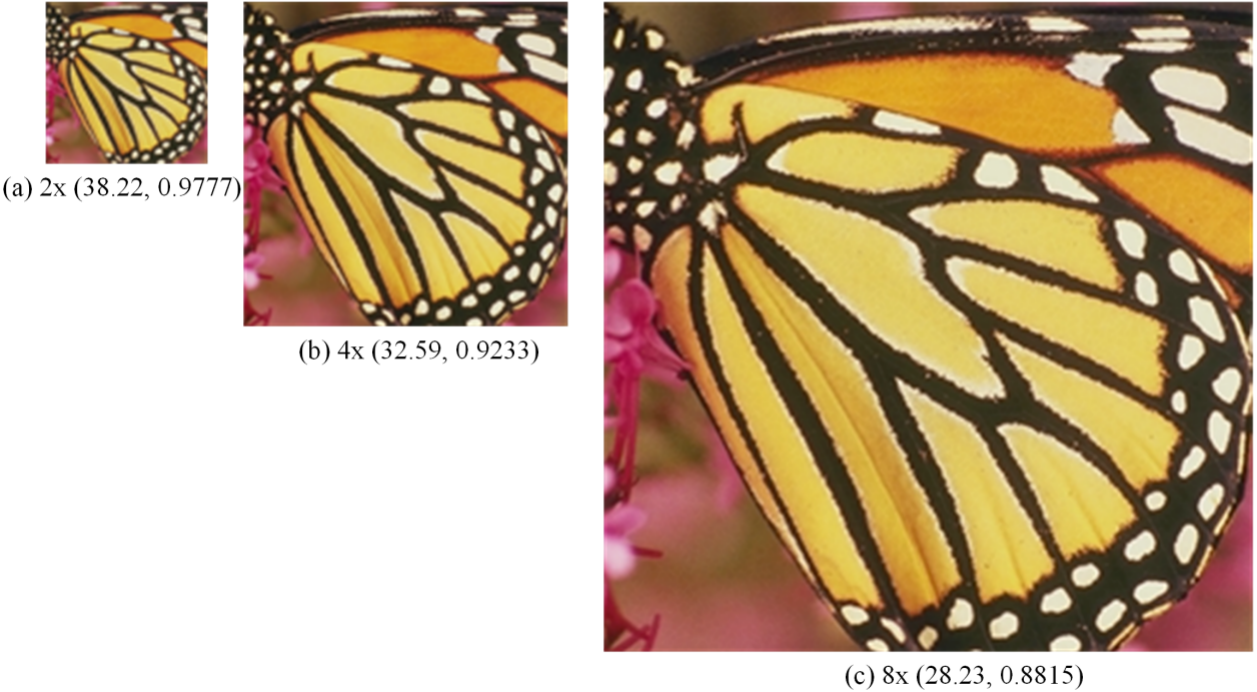
SR images of butterfly. The 2 ×, 4 × and 8 × SR images of butterfly in Set5 reconstructed by the proposed method. Image source credit: Bevilacqua et al. (2012). Dataset: https://people.rennes.inria.fr/Aline.Roumy//results/SR_BMVC12.html.

In Fig. 7, the SR images generated by bicubic and the first three methods are not very natural, and the SR images generated by the latter two methods and our method are more natural. In Fig. 8, with a window in a high-rise building in the generated SR image, the image generated by our method is more natural and clear with better edge performance, and for the one window in a tall building in Fig. 8, the details of the image reconstruction are improved using the texture loss function. Figure 9 shows the 2 ×, 4 × and 8 × SR images of the butterfly in Set5 reconstructed by the proposed method. Among them, the LR image used for reconstruction is a 32 ×32 image obtained by resizing the LR butterfly image in set5, and the SR images of 64 ×64, 128 ×128, and 256 ×256 are obtained by the proposed method. Their PSNR and SSIM are (38.22, 0.9777), (32.59, 0.9233), and (28.23, 0.8815), respectively. It can be seen that the image quality decreases rapidly as the number of reconstructions increases.

Table 1 shows the average performance of several methods on the four datasets. The proposed method performs best on 2xSR images and achieves the best effect on the four datasets. Our proposed method is not the best in terms of image evaluation indicators PSNR and SSIM, but it is the best in terms of detail and texture.

To further verify the method proposed in this paper, Table 2 gives the parameters and average testing time of the method proposed in this paper and other deep learning models.

As can be seen from Table 2, the number of parameters of the proposed model in this paper is only more than the number of RNAN parameters, and the average test speed is the fastest. The proposed method achieves a good balance between reconstruction performance and model complexity.

## Conclusions

Garesat-Net SR was proposed to reconstruct realistic textures, edges and details by obtaining high visual quality. Gaussian enhancement block, spatial attention module and channel attention module were applied. The Gaussian enhancement block can enhance the edge information in the feature map, so that the final SR image is not blurred at the edges. During the learning process, the feature maps of LR images are obtained and enhanced by convolutional layer learning. Some feature maps contain only a small amount of information, while some learn most of the information of the LR image. Channel attention enhances the weights of the feature maps with more information, and the spatial attention module enhances the weights of the area containing important information in the feature map.

In the training process, the use of the pixel loss function ensures more similarity in color and structure. SR images score higher in PSNR and SSIM. The use of the texture loss function results in SR images with more detailed structure, more realistic texture and more natural details. Numerous experiments show that Garesat SR surpasses the existing models and achieves the most advanced performance. Finally, the proposed method and the five most advanced deep learning SR methods are tested on four datasets: Set 5, Set14, BSD100, and Urban100. Experimental results show that the PSNR and SSIM of the SR images obtained by the proposed method are not optimal during testing, but the obtained images are more natural and closer to HR images in terms of details and edges. After enlarging the details, we can also see that there are differences between SR images and HR images, which means that the SR algorithm needs further improvement, which will be our future work.

**Table 2:** Deep learning model parameter comparison.

	RNAN	LatticeNet	SRGAN	CMG	Proposed
Number of parameters	69087	909283	710343	1883045	793899
Average of test time	2.8	2.63	1.03	1.01	0.85

##  Supplemental Information

10.7717/peerj-cs.1182/supp-1 Supplemental Information 1 Code of the GaresatNet

## Additional Information and Declarations

### Data Availability

The following information was supplied regarding data availability: The data is available from: - In Proceedings British Machine Vision Conference 2012. Pages 135.1–135.10.
http://dx.doi.org/10.5244/C.26.135
- Zeyde, R., Elad, M., Protter, M. (2012). On Single Image Scale-Up Using Sparse-Representations. In: , et al. Curves and Surfaces. Curves and Surfaces 2010. Lecture Notes in Computer Science, vol 6920. Springer, Berlin, Heidelberg. https://doi.org/10.1007/978-3-642-27413-8_47
- D. Martin, C. Fowlkes, D. Tal and J. Malik, “A database of human segmented natural images and its application to evaluating segmentation algorithms and measuring ecological statistics,” Proceedings Eighth IEEE International Conference on Computer Vision. ICCV 2001, 2001, pp. 416-423 vol.2, doi: 10.1109/ICCV.2001.937655. - J. -B. Huang, A. Singh and N. Ahuja, “Single image super-resolution from transformed self-exemplars,” 2015 IEEE Conference on Computer Vision and Pattern Recognition (CVPR), 2015, pp. 5197-5206, doi: 10.1109/CVPR.2015.7299156. - Kaggle: https://www.kaggle.com/datasets/msahebi/super-resolution; Nie, Wenfang (2022): BSD100, Set5, Set14, Urban100. figshare. Dataset. https://doi.org/10.6084/m9.figshare.21586188.v1
The code is available at Github: https://github.com/jbhuang0604/SelfExSR, DOI: 10.1109/CVPR.2015.7299156.
